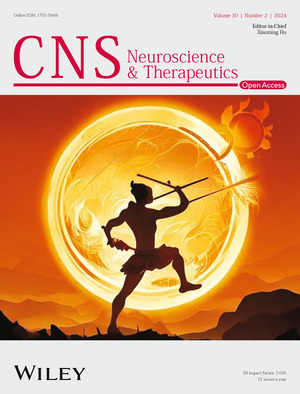# Additional Cover

**DOI:** 10.1111/cns.14665

**Published:** 2024-02-29

## Abstract

The cover image is based on the Original Article *Electroacupuncture ameliorates neuroinflammation by inhibiting TRPV4 channel in ischemic stroke* by Xueqi Ren et al., https://doi.org/10.1111/cns.14618.